# The challenge of etiologic diagnosis of subacute and chronic meningitis: an analysis of 183 patients

**DOI:** 10.1017/S0950268824001225

**Published:** 2024-10-10

**Authors:** Mahboubeh Haddad, Fereshte Sheybani, Matin Shirazinia, Farzaneh Khoroushi, Zahra Baghestani

**Affiliations:** 1 Department of Infectious Diseases and Tropical Medicine, Faculty of Medicine, Mashhad University of Medical Sciences, Mashhad, Iran; 2 Faculty of Medicine, Mashhad University of Medical Sciences, Mashhad, Iran; 3 Department of Radiology, Faculty of Medicine, Mashhad University of Medical Sciences, Mashhad, Iran; 4 Department of Neurology, Faculty of Medicine, Mashhad University of Medical Sciences, Mashhad, Iran

**Keywords:** Brucella meningitis, etiologic diagnosis, subacute and chronic meningitis, tuberculous meningitis, Prognosis

## Abstract

Subacute and chronic meningitis (SCM) presents significant diagnostic challenges, with numerous infectious and non-infectious inflammatory causes. This study examined patients aged 16 and older with SCM admitted to referral centers for neuroinfections and neuroinflammations in Mashhad, Iran, between March 2015 and October 2022. Among 183 episodes, tuberculous meningitis was the most common infectious cause (46.5%), followed by Brucella meningitis (24.6%). The cause of SCM was definitively proven in 40.4%, presumptive in 35.0%, and unknown in 24.6% of cases. In-hospital mortality was 14.4%, and 30.5% of survivors experienced unfavorable outcomes (Glasgow Outcome Scale 2–4). Patients with unknown causes had a significantly higher risk of death compared to those with presumptive or proven diagnoses (risk ratio 4.18). This study emphasizes the diagnostic difficulties of SCM, with one-quarter of cases remaining undiagnosed and over one-third having only a presumptive diagnosis. Improving diagnostic methods could potentially enhance prognosis and reduce mortality.

## Introduction

Meningitis can be classified according to its underlying cause or the duration of the illness. Based on the time course, it is categorized as acute, subacute, or chronic [1]. Chronic meningitis is characterized by meningeal disease with cerebrospinal fluid (CSF) inflammation lasting 4 weeks or more, without clinical improvement or with clinical worsening [2]. While there is less consensus on the definition of subacute meningitis, it is often described as meningitis that persists for several weeks, or more specifically, for a duration between 5 days and 1 month [3, 4].

Chronic meningitis is associated with considerable morbidity and mortality [5]. Unlike acute meningitis, subacute and chronic meningitis (SCM) have a more gradual onset and a longer duration. In many cases, it can take several weeks or even months from the initial symptoms to reach a diagnosis [6]. The actual incidence and prevalence of chronic meningitis differ significantly across various regions and populations but the lack of comprehensive population-based studies makes it difficult to accurately determine its epidemiology. Anecdotal evidence indicates that chronic meningitis accounts for a small percentage of all meningitis cases, generally estimated to be between 5% and 10% [5]. Despite its relatively lower prevalence compared to acute meningitis, chronic meningitis imposes significant clinical and economic burdens due to extended hospital stays, repeated diagnostic testing, and the possibility of long-term complications.

The causes of SCM are diverse. Despite its clinical importance, SCM is relatively underexplored in medical literature, with a limited number of comprehensive studies addressing its diverse causes, diagnostic methods, and treatment options. Most existing literature on this topic comprises case reports and a few retrospective case series from individual centres. As a result, selection, publication, and ascertainment biases significantly limit the usefulness of this literature in evaluating the distribution of patients by specific etiologic diagnosis [1]. Consequently, the current literature may not offer a thorough or representative view of the disease, making it difficult for clinicians to make well-informed decisions about diagnosis and treatment.

SCM presents a complex diagnostic challenge, involving both infectious and non-infectious causes. Identifying the specific cause of chronic meningitis is difficult, with no definitive etiology found in at least one-third of cases [7]. This challenge is highlighted by a Mayo Clinic study, which revealed that despite performing over 2,000 tests on the cerebrospinal fluid (CSF) of 37 patients with chronic idiopathic meningitis, a definitive diagnosis was made in fewer than half of the cases [8].

The limited number of studies available on chronic meningitis underscore the need for further investigation into this complex condition to improve our understanding, diagnostic accuracy, and therapeutic outcomes. Here, we describe the characteristics of meningitis in a population of adults with SCM. We analyzed the cause-specific diagnosis of SCM and the association of the level of certainty about the etiologic diagnosis of meningitis with the clinical outcome.

## Methods

Our study was a cross-sectional investigation aimed at assessing the association between the level of certainty regarding the etiologic diagnosis of SCM and the clinical outcomes of patients. We analyzed data from patients aged 16 years and older with SCM who were admitted to one of the two main university hospitals in Mashhad, Iran, between March 2015 and October 2022. These hospitals are the main referral centres for adults with community-acquired neuroinfections and neuroinflammations in the region. Located in Northeast Iran, Mashhad is the second most populous city in the country, with a population of approximately 3.5 million.

For patients admitted from March 2015 to September 2019, we reviewed their medical records and discharge letters. For those admitted from October 2019 to October 2022, who were included in our prospective cohort of community-acquired central nervous system (CNS) infections, data were collected prospectively via an online patient registration form. Data regarding patient history, symptoms and signs upon admission, laboratory findings, neuroimaging results, outcomes, etiologic diagnosis, and the level of certainty about the etiologic diagnosis were collected.

SCM was defined as meningitis lasting for weeks or months without clinical improvement or with clinical worsening. The definition excludes meningitis occurring concurrently with mass lesions of the CNS, meningitis associated with previously diagnosed cases of a systemic disease known to cause meningitis, and meningitis associated with trauma or following neurosurgical procedures [3]. Additionally, cases of viral meningitis, which commonly cause subacute meningitis, were not included in our study.

**Figure 1.**
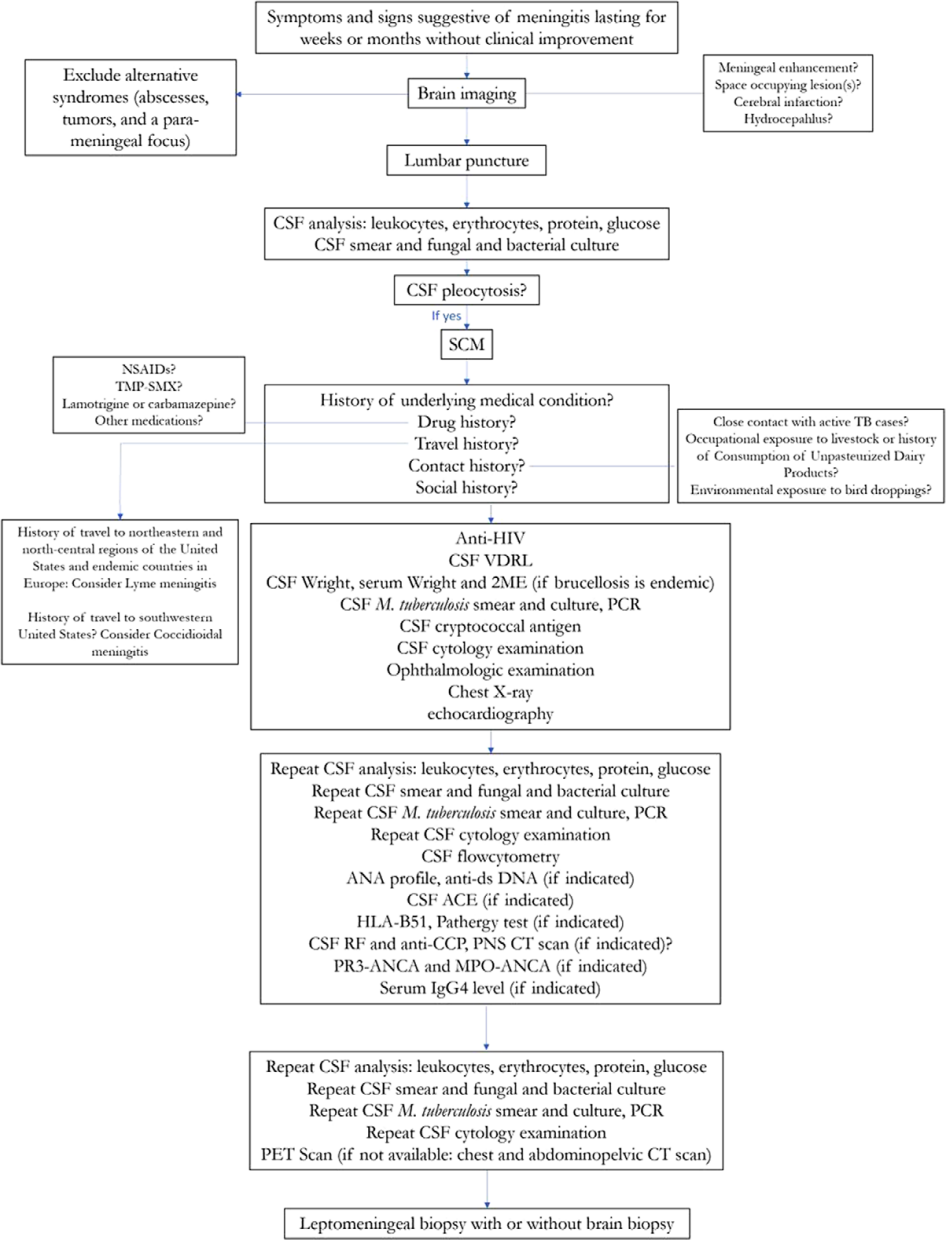
Diagnostic algorithm for identifying subacute and chronic meningitis and its etiologic cause. ACE, angiotensin-converting enzyme; ANA, antinuclear antibody; ANCA, anti-neutrophil cytoplasmic antibody; anti-CCP, anti-cyclic citrullinated peptide; anti-dsDNA, anti-double stranded deoxyribonucleic acid; CSF, cerebrospinal fluid; HIV, human immunodeficiency virus; HLA, Human leukocyte antigen; IgG4, immunoglobulin G4; MPO, myeloperoxidase; *Mycobacterium tuberculosis*, *M. tuberculosis*; NSAIDs, non-steroidal anti-inflammatory drugs; PCR, polymerase chain reaction; PET scan, positron emission tomography; PNS CT scan, paranasal sinuses computed tomography scan; PR3, proteinase 3; RF, rheumatoid factor; SCM, subacute and chronic meningitis; TB, tuberculosis; TMP-SMX, trimethoprim-sulfamethoxazole; VDRL, venereal disease research laboratory test; 2ME, 2-Mercaptoethanol

The diagnostic algorithm that was used for identifying SCM and its etiologic cause is shown in Figure 1.

The level of diagnostic certainty reflects how well the etiologic diagnosis has been identified. All patients who presented with the syndrome of SCM were initially considered as possible cases of infectious, autoimmune, neoplastic, or chemical/drug-induced meningitis. Following a thorough diagnostic evaluation, they were classified into one of the three following levels of diagnostic certainty: proven, presumptive, and unknown cause-specific diagnoses.

In the category of proven diagnosis, the etiology of meningitis was identified by using culture-based, molecular-based, or serologic tests on the CSF specimens or brain or meningeal tissues, or relevant extra-neural tissues or specimens. Or in cases of non-infectious meningitis if the findings met the diagnostic criteria for a specific etiology.

In the category of presumptive diagnosis, a high level of certainty about a specific cause of meningitis was achieved, but it did not meet the criteria for a proven diagnosis. In these instances, the treating physician presumed a specific cause of meningitis based on a combination of clinical, laboratory, and imaging characteristics. Alternative causes were considered unlikely or excluded, and the patient was treated or managed according to this presumed etiologic diagnosis.

In the category of unknown cause, the treating physician was uncertain about the cause-specific diagnosis because the available findings did not hold an opinion or conclusion about a specific etiology.

We used the Glasgow outcome scale (GOS) for scoring the clinical outcome that was assessed based on the patient’s condition at discharge from the hospital. A favourable outcome was defined as a score of 5 and an unfavourable outcome as a GOS score of 2–4.

Statistics: Continuous data were described with medians and interquartile range (percentile 25 to percentile 75) and categorical variables with frequency and percentage. Fisher’s exact test and chi-square tests were used for categorical variables, as appropriate. The risk ratio (RR), along with its corresponding 95% confidence interval (95% CI), was used as the effect size to illustrate the association between clinical and paraclinical characteristics and both mortality and unfavourable outcomes. A *p*-value <0.05 was considered statistically significant.

Ethics: This study was approved by the Ethics Committee of Mashhad University of Medical Sciences under project number 970030 and the ethics code of IR.MUMS.MEDICAL.REC.1397.292. Informed consent was obtained during admission from participating patients.

## Results

Overall, 183 episodes of SCM were diagnosed, including 72 (39.3%) episodes of meningitis lasting for at least 4 weeks and the remaining episodes with subacute meningitis.

The median age of the patients was 37 (percentile 25 to percentile 75, 26 to 55) years, and 104 (56.8%) patients were male. The study sample consisted of 22 (12.0%) elderly patients, defined as individuals aged 65 years or older. Twenty-one (11.7%) episodes occurred in immunocompromised patients including three (1.7%) individuals with HIV/AIDS (Table 1).

**Table 1.** Characteristics of patients with subacute and chronic meningitis

Variables	Total (*n* = 183)
Age (years), median (percentile 25 to percentile 75)	37 (26 to 55)
Elderly (≥65 years), *n* (%)	22/183 (12)
Gender (male), *n* (%)	104/183 (56.8)
Underlying comorbidities, *n* (%)	
Immunocompromised	21/179 (11.7)
Diabetes mellitus	13/178 (7.3)
Cancer	4/179 (2.2)
Rheumatologic disorders	7/178 (3.9)
HIV/AIDS	3/179 (1.7)
CKD/ESRD	3/179 (1.7)
Organ transplant	2/179 (1.1)
Clinical manifestations, *n* (%)	
Headache	129/168 (76.8)
Fever	137/168 (81.6)
Seizures	26/176 (14.8)
Altered consciousness	94/180 (52.2)
Neck stiffness	56/145 (38.6)
FNDs	52/153 (34.0)
Classic triad of meningitis^a^, *n* (%)	19/157 (12.1)
Symptom duration (days), median (percentile 25 to percentile 75)	20 (9 to 40)
Neuroimaging findings, *n* (%)	
Normal	95/142 (66.9)
Hydrocephalus	41/179 (22.9)
Meningeal enhancement	31/163 (19.0)
Cerebral infarction	18/161 (11.2)
Microabscess/granuloma formation	15/164 (9.2)
Laboratory features	
CSF leukocytes (count/μL), median (percentile 25 to percentile 75)	87.5 (32.5 to 225)
CSF lymphocyte (count/μL), median (percentile 25 to percentile 75)	56.8 (19 to 178.5)
Lymphocyte	129/171 (75.4)
Predominance^b^, *n* (%)	
CSF protein (mg/dL), median (percentile 25 to percentile 75)	106 (62 to 173)
CSF glucose (mg/dL), median (percentile 25 to percentile 75)	40 (24 to 57)
Severe hypoglycorrhachia (glucose<10 mg/dL), *n* (%)	19/180 (10.6)
Severe hyperproteinorrhachia (protein≥500 mg/dL), *n* (%)	9/179 (5.0)
Length of hospital stay (days), median (percentile 25 to percentile 75)	17 (10 to 27)
Unfavourable outcome (at discharge), *n* (%)	73/180^c^ (40.6)
In–hospital mortality, *n* (%)	26/183 (14.2)

AIDS, acquired immunodeficiency syndrome; CKD/ESRD, end-stage renal disease/chronic kidney disease; CSF, cerebrospinal fluid; FNDs, focal neurologic deficits; HIV, human immunodeficiency virus.

a Characterized by fever, headache, and neck stiffness.

b Defined as comprising more than 50% lymphocytes in cerebrospinal fluid.

c Three patients left the hospital against medical advice.

The most common clinical manifestation of meningitis was fever in 137 (81.6%) of 168 patients, followed by headache in 129 (76.8%) of 168, altered consciousness in 94 (52.2%) of 180, and neck stiffness in 56 (38.6%) of 145. Neurological deficits were recorded in 52 (34%) of 153 patients and seizures in 26 (14.8%) of 176 episodes. The classic triad of meningitis, characterized by fever, headache, and neck stiffness, was identified in 19 (12.1%) of 157 cases. CSF analysis revealed a median CSF leukocyte count of 87.5/μL (percentile 25 to percentile 75, 32.5 to 225) with lymphocyte predominance, defined as comprising more than 50% lymphocytes, noted in 129 (75.4%) of 171 episodes.

**Figure 2.**
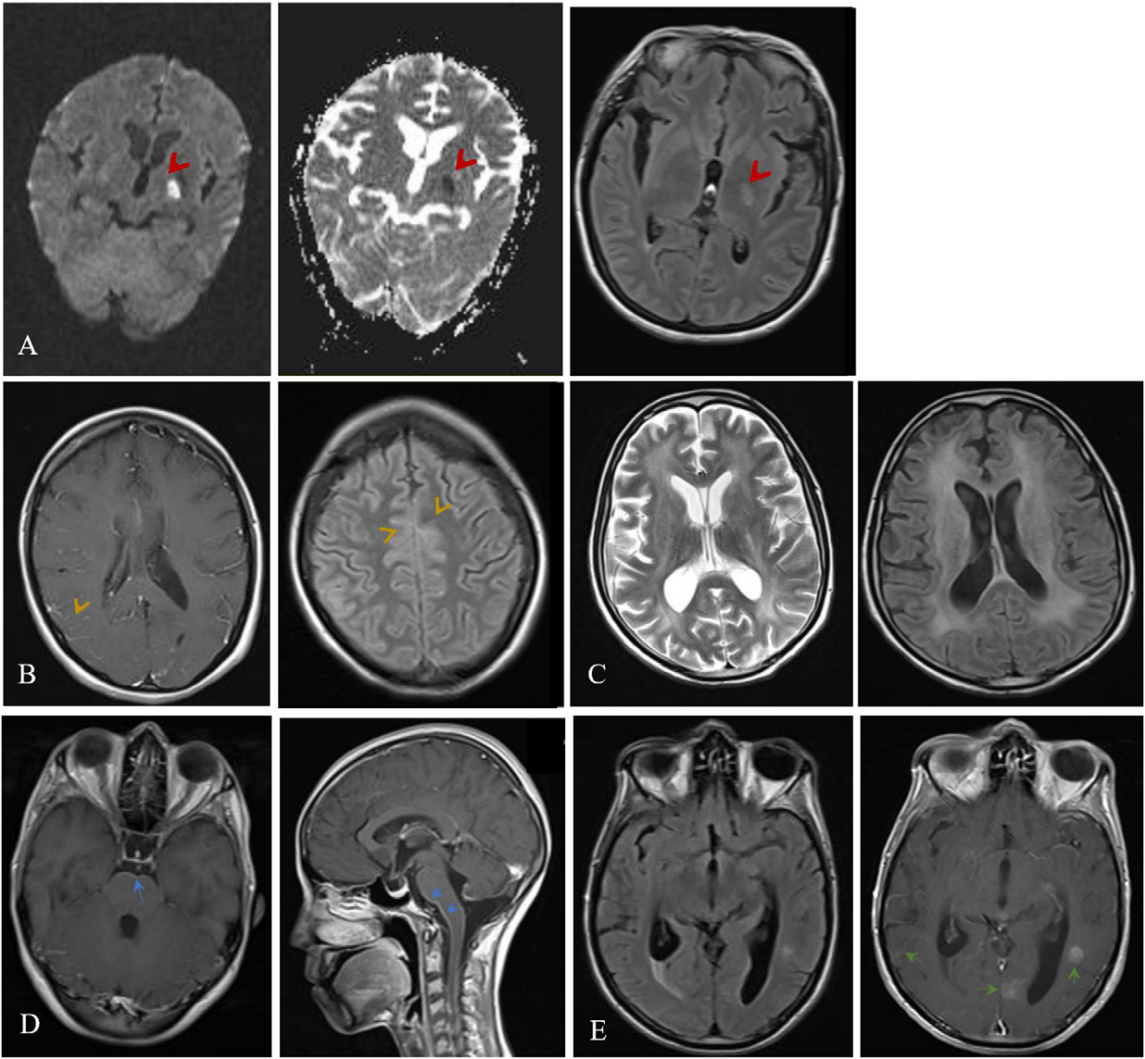
Abnormal neuroimaging findings in patients with subacute and chronic meningitis. (a) A 33-year-old woman with CNS tuberculosis complicated with brain infarct. Diffusion-weighted image (DWI) and ADC show restricted diffusion in left basal ganglia, hallmark feature of acute infarction (left images) and FLAIR image shows high signal intensity in left basal ganglia corresponding to the lesion seen on DWI and ADC. (b) A 19-year-old woman with CNS tuberculosis diagnosed early after a stillbirth. Post contrast T1 weighted image shows leptomeningeal enhancement and FLAIR image demonstrates sulcal hyperintensity (CSF dirty sign). (c) A 36-year-old man, new case of HIV infection with cryptococcal meningoencephalitis and HIV-associated leukoencephalopathy who died before antifungal treatment was started. T2 weighted and FLAIR images show hydrocephalus and white matter hyperintensity. (d) A 27-year-old woman with Brucella meningitis presented with multiple cranial nerve palsy. Post contrast T1 weighted images show leptomeningeal enhancement around brain stem. (e) A 46-year-old man with carcinomatous meningoencephalitis presented with multiple cranial nerve palsy. Post contrast T1 weighted image demonstrates leptomeningeal enhancement and multiple enhancing lesions.

Brain computed tomography (CT) scan and brain magnetic resonance imaging (MRI) were normal in 68 (41.2%) of 165 and 44 (31.2%) of 141 episodes, respectively. The most common abnormal findings on neuroimaging in the descending order of frequency were ventricular enlargement in 41 (22.9%) of 179 cases, meningeal enhancement in 31 (19.0%) of 163, cerebral infarct in 18 (11.2%) of 161, and micro abscess/granuloma formation in 15 (9.2%) of 165 (Figure 2).

Proven cause-specific diagnosis was made in 74 (40.4%) of 183 episodes. In 64 (35.0%) cases, the etiologic diagnosis remained presumptive, and in 45 (24.6%) episodes, no presumption could be made about the etiologic cause of meningitis. The most common presumed or proven cause of meningitis was *Mycobacterium tuberculosis* in 85 (46.5%) of the episodes, followed by Brucella species in 45 (24.6%), carcinomatous meningitis in 3 (1.6%), cryptococcal meningitis and neurobehçet disease, each in 2 (1.1%) and neuropsychiatric lupus in one. Among those with proven diagnosis, Brucella was the most common causative agent (*n* = 43, 58.1%), followed by *M. tuberculosis* (*n* = 24, 32.4%), autoimmune causes (*n* = 3, 4.1%), and Cryptococcal, and carcinomatous meningitis (*n* = 2, 2.7% each). Regarding presumptive etiology, *M. tuberculosis* was the predominant cause in 61 patients (95.3%), followed by Brucella in two patients (3.1%), and carcinomatous meningitis in one patient.

Seventy-three (40.6%) patients experienced unfavourable outcomes at discharge from the hospital including 26 (14.4%) cases of in-hospital mortality.

**Figure 3.**
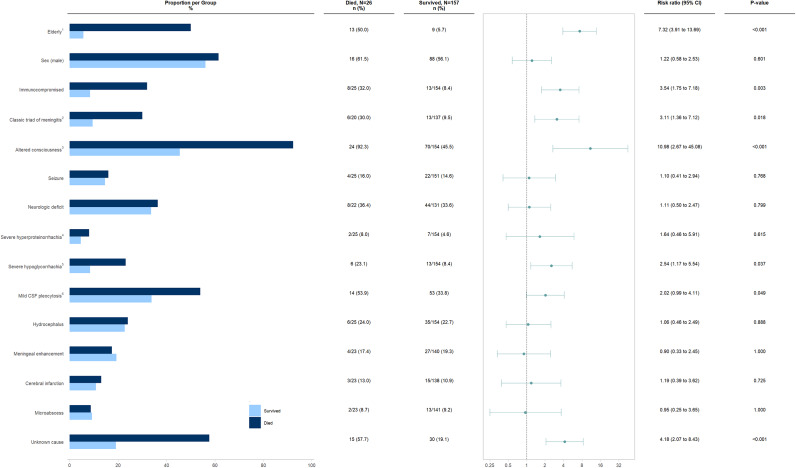
Univariable analysis of the association between various clinical and paraclinical characteristics and the risk of in-hospital mortality in patients with subacute and chronic meningitis. ^1^Defined as ≥65 years; ^2^Characterized by fever, headache, and neck stiffness; ^3^Defined as Glasgow Coma Scale<15; ^4^Defined as protein≥500 mg/dL; ^5^Defined as glucose<10 mg/dL; ^6^Defined as comprising more than 50% lymphocytes in CSF

Univariable analysis showed that the risk of in-hospital death was 4.18 times significantly higher in the group with meningitis of unknown cause compared to those with a proven or at least presumed cause of meningitis (*p*-value: <0.001; RR: 4.18; 95% CI 2.07 to 8.43). Older age (*p*-value: <0.001; RR: 7.32; 95% CI 3.91 to 13.69), underlying immunosuppression (*p*-value: 0.003; RR: 3.54; 95% CI 1.75 to 7.18), presence of the classic triad of meningitis (*p*-value: 0.018; RR: 3.11; 95% CI 1.36 to 7.12), decreased level of consciousness on presentation (*p*-value<0.001; RR: 10.98; 95% CI 2.67 to 45.08), severe hypoglycorrhachia (*p*-value: 0.037; RR: 2.54; 95% CI 1.17 to 5.54), and a mild CSF leukocyte of less than 50/μL (*p*-value 0.049; RR: 2.02; 95% CI 0.99 to 4.11) were significantly associated with a higher risk of in-hospital death (Figure 3).

**Figure 4.**
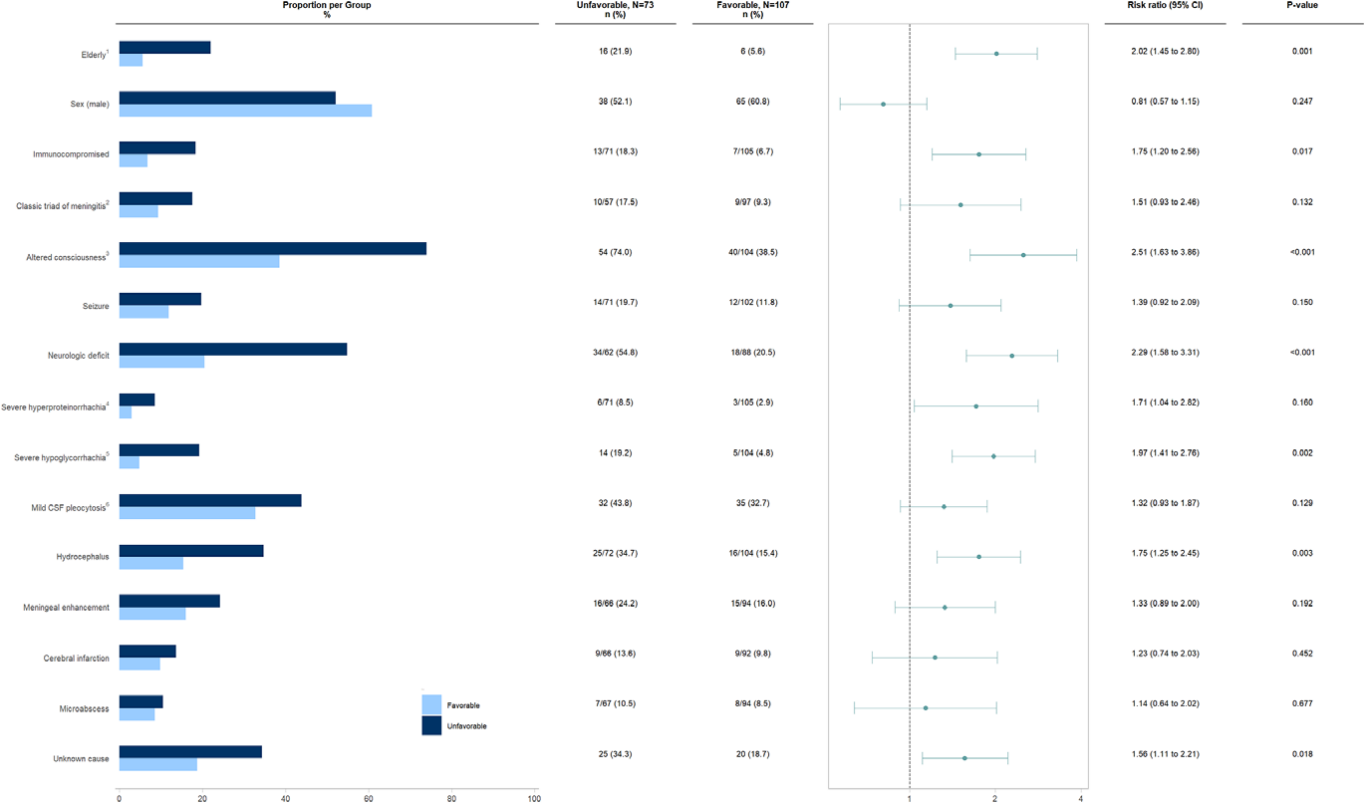
Univariable analysis of the association between various clinical and paraclinical characteristics and the risk of unfavourable outcome at hospital discharge in patients with subacute and chronic meningitis. ^1^Defined as ≥65 years; ^2^Characterized by fever, headache, and neck stiffness; ^3^Defined as Glasgow Coma Scale<15; ^4^Defined as protein≥500 mg/dL; ^5^Defined as glucose<10 mg/dL; ^6^Defined as comprising more than 50% lymphocytes in CSF.

In the analysis of various clinical and paraclinical features’ impact on unfavourable outcomes, older age (*p*-value: 0.001; RR: 2.02; 95% CI 1.45 to 2.80), underlying immunosuppression (*p*-value: 0.017; RR: 1.75; 95% CI 1.20 to 2.56), altered consciousness on presentation (*p*-value: <0.001; RR: 2.51; 95% CI 1.63 to 3.86), neurological deficits (*p*-value: <0.001; RR: 2.29; 95% CI 1.58 to 3.31), severe hypoglycorrhachia (*p*-value: 0.002; RR: 1.97; 95% CI 1.41 to 2.76), hydrocephalus (*p*-value: 0.003; RR: 1.75; 95% CI 1.25 to 2.45), and unknown etiology (*p*-value: 0.018; RR: 1.56; 95% CI 1.11 to 2.21) were significantly associated with unfavourable outcomes at hospital discharge (Figure 4).

## Discussion

To our knowledge, this is the largest study to analyze the characteristics, cause-specific diagnoses, and clinical outcomes of patients with SCM. Our findings reveal that when patients with SCM were admitted for diagnostic evaluation, the cause remained unidentified in 25% of cases and was presumptively diagnosed in 35%. Those with meningitis of unknown etiology had a significantly higher risk of in-hospital death, being 4.2 times more likely to die compared to patients with a confirmed or presumptive diagnosis. Among those who survived to discharge, approximately 30% faced adverse outcomes. Factors linked to a higher risk of in-hospital death included older age (relative risk [RR]: 7.32), altered consciousness upon presentation (RR: 10.98), the presence of the classic triad of meningitis (RR: 3.11), underlying immunosuppression (RR: 3.54), severe hypoglycorrhachia (RR: 2.54), and a cerebrospinal fluid leukocyte count of less than 50/μL (RR: 2.02).

Information on the causes of SCM is limited and varies significantly across different studies. The available literature offers only a partial view of the diverse causes, which include infectious, autoimmune, and neoplastic origins, with prevalence rates differing by region and country [9]. Globally, tuberculous and cryptococcal meningitis are recognized as the most common types of chronic meningitis [10]. In our study, tuberculous meningitis emerged as the most frequent cause of SCM, followed by Brucella and carcinomatous meningitis. Among cases with microbiologically confirmed diagnoses, Brucella was the predominant pathogen. These findings align with previous research from Tehran, Iran, where tuberculous, Brucella, and carcinomatous meningitis were identified as leading causes of SCM [11, 12]. However, detailed information on chronic meningitis remains sparse. For instance, a study conducted in Bangkok, involving 114 patients with a high prevalence of HIV, found tuberculous meningitis in 37% of cases and cryptococcal meningitis in 54% [13]. Similarly, a 16-year study in New Zealand involving 83 patients without predisposing conditions reported 40% with tuberculous meningitis and 17% responding to empiric anti-TB therapy [14]. A more recent multinational study on community-acquired CNS infections (2012–2014) found that chronic disease was present in 4.3% of 2,583 episodes. Among neuroinfections, neurosyphilis, Brucella meningitis, neuroborreliosis, and CNS tuberculosis were significantly more likely to present with chronic courses. Although cryptococcal meningitis represented only 2.2% of cases with chronic CNS disease, it was notably the most frequent community-acquired CNS infection among HIV-positive individuals [15]. Despite these findings, the overall understanding of chronic meningitis remains incomplete, underscoring the need for further research to fully elucidate its epidemiology and clinical characteristics.

Regional variations in socioeconomic factors, immunization coverage, migration patterns, and the prevalence of autoimmune disorders such as systemic lupus erythematosus, sarcoidosis, and Behçet’s disease may contribute to the differences observed in the etiological diagnoses reported in our study compared to those from other countries [9]. While infectious meningitis remains the predominant cause of SCM in our study and most similar reports, some more developed nations have noted a decline in the incidence of infectious meningitis compared to earlier data [16]. For example, a prospective study conducted at an academic centre in Amsterdam from 2012 to 2015, which reviewed 363 suspected CNS infection episodes, found that cryptococcal meningitis, tuberculous meningitis, and neurosarcoidosis each accounted for three cases among 125 neuroinfections and neuroinflammations [17]. The higher proportion of infectious meningitis in our study likely reflects the elevated prevalence of infectious diseases such as tuberculosis and brucellosis in our community and our study’s exclusion of autoimmune meningitis in patients already diagnosed with systemic autoimmune disorders known to cause meningitis. CNS involvement as the initial manifestation of a systemic autoimmune disorder is rare in previously healthy individuals. However, sarcoidosis is an exception, with neurological symptoms presenting in 50%–70% of cases of neurosarcoidosis [18]. A meta-analysis of 1,088 patients with neurosarcoidosis found that only 31% had systemic disease at the time of presentation, while 84% developed systemic manifestations over time [19].

Certain infectious organisms that are either endemic or newly emerging and can cause SCM may also be responsible for a significant proportion of meningitis cases in the regions where they are prevalent. For example, coccidioidomycosis is a notable cause in the southwestern United States, histoplasmosis frequently occurs in the central and eastern United States – particularly around the Ohio and Mississippi River valleys [20] – and Lyme disease is commonly seen in the northeastern, mid-Atlantic, and upper midwestern United States as well as in central Europe and Scandinavia [21].

The influence of underlying comorbidities on chronic meningitis is considerable. The prevalence of these comorbidities in chronic meningitis patients can vary based on geographic and socioeconomic factors [10]. For instance, in regions with high HIV/AIDS rates, cryptococcal meningitis often emerges as the leading cause of chronic meningitis, sometimes resulting in more deaths than tuberculosis [22]. A study in Bangkok, Thailand, where HIV prevalence is high, found cryptococcal meningitis to be the most common cause of chronic meningitis, occurring in 54% of cases [13]. Similarly, a study in Addis Ababa, Ethiopia, between 2003 and 2004, found 15% of CSF samples positive for cryptococcal antigen, with about half of these patients having HIV/AIDS [23]. In contrast, our study revealed that 12% of patients with SCM were immunocompromised, with only 2% having HIV/AIDS, and cryptococcal meningitis accounted for 1.1% of SCM cases. Additionally, the rate of neoplastic meningitis can differ by population; for example, in a Buenos Aires study of 70 patients with SCM from 2007 to 2017, neoplastic meningitis was found in 70% of cases [9].

Our study highlights the poor outcomes associated with SCM, showing that 41% of patients experienced unfavourable outcomes at hospital discharge, including 14% who died during their stay. Patients with SCM of unknown cause had nearly a 60% higher risk of unfavourable outcomes at discharge (relative risk: 1.56) and a fourfold higher risk of death (relative risk: 4.18) compared to those with a confirmed or presumptive cause-specific diagnosis. Factors significantly associated with increased in-hospital mortality included older age, immunocompromised status, the presence of the classic triad of meningitis, altered consciousness at presentation, severe hypoglycorrhachia, and low cerebrospinal fluid (CSF) leukocyte count (<50/μL). For unfavourable outcomes at discharge as measured by the Glasgow Outcome Scale (GOS), significant predictors included older age, underlying immunosuppression, altered consciousness at presentation, neurological deficits, severe hypoglycorrhachia, and hydrocephalus.

Although challenging, diagnosing the specific cause of chronic meningitis is essential for selecting the most effective treatment and providing accurate prognostic information [5]. The complexity of making the etiological diagnosis of chronic meningitis can be compared to the difficulty in identifying the cause of fever of unknown origin (FUO), of course, here in the CNS. As the list of potential causes of meningitis expands, the cost of diagnostic evaluations is rising [16]. Specific clues from patient history, physical exams, and laboratory results can help narrow down the possibilities, but it is not always feasible to sequence diagnostic tests based on their prevalence and these clues alone, as this might lead to delays in diagnosis and treatment [16]. Neuroimaging primarily helps rule out alternative conditions such as abscesses, tumours, or infections in the paranasal sinuses or paravertebral areas. Most patients with chronic meningitis have either normal imaging or nonspecific findings [3]. In our study, 67% of neuroimaging results were normal, 23% showed hydrocephalus (the most common abnormal finding), 19% had meningeal enhancement, and 11% presented with associated cerebral infarctions.

Our study also highlights a significant gap in understanding the etiology of SCM. The markedly higher risk of in-hospital mortality and adverse outcomes in patients with meningitis of unknown origin underscores the urgent need for prompt and accurate diagnosis. Early identification is crucial for implementing effective treatment strategies and improving patient outcomes. These results emphasize the necessity for further research and investment in advanced diagnostic methods to improve both the accuracy and speed of diagnoses. Currently, traditional diagnostic approaches for SCM often fall short, as they frequently fail to identify the specific cause in a substantial number of patients.

In our study, only 40% of patients were found to have a microbiologically confirmed, cause-specific diagnosis. The development of new noninvasive diagnostic methods, such as multiplex PCR, 16S, 18S, and 28S ribosomal RNA PCR, and metagenomic next-generation sequencing (mNGS), offers promising advancements for detecting pathogens and holds potential for diagnosing complex cases of chronic meningitis with a broad range of possible causes [7]. While mNGS is considered a more ‘unbiased’ and hypothesis-free diagnostic approach, its use in chronic meningitis is still limited, with evidence primarily from a few case reports and a recent case series of patients with idiopathic chronic meningitis. Furthermore, analyzing mNGS results requires meticulous assessment to distinguish true pathogens from environmental contaminants, as misinterpretation can lead to incorrect associations of meningitis with organisms later identified as laboratory contaminants [24]. The overall yield of leptomeningeal biopsy, with or without brain biopsy, is generally low and depends on the presence of enhancing lesions on brain MRI. Many chronic meningitis cases involve nonspecific inflammation, making a definitive diagnosis difficult even after a biopsy [25]. Due to its invasive nature, biopsy should be reserved for patients with worsening clinical conditions or those undergoing neurosurgical procedures [5].

Our study has several limitations. We acknowledge that the epidemiology of chronic meningitis varies significantly by geographic region. Additionally, the incidence, manifestations, severity, and outcomes of SCM are influenced not only by the causative agent but also by the patient’s underlying conditions, such as immune status, age, and prior sensitization. As a result, our study may be subject to selection bias due to the specific study population and local referral patterns, which could impact the distribution of causative agents and their clinical outcomes. Furthermore, we did not follow patients beyond their hospital discharge, leaving the long-term outcomes for those who might have experienced changes in their neuropsychiatric condition due to meningitis or its treatment after discharge unknown. Additionally, regarding the analysis of the relationship between various clinical and paraclinical features and mortality, we only employed univariable analysis without adjusting for confounding factors, thereby reducing the certainty of our results. The reason we refrained from employing multivariable analysis was the limited number of positive outcome patients (*n* = 26), which, if adjusted for various independent variables, would have led to sparse data. Lastly, we did not use 16S rRNA or metagenomic next-generation sequencing (mNGS) in the diagnostic evaluation of our patients. Consequently, the proportion of patients with cause-specific diagnoses in our study may not reflect the true number of SCM patients who could have received an etiological diagnosis with the use of these newer tests. However, it shows that there is still a lot of room for improvement in cause-specific diagnosis of SCM and patient outcomes.

## Conclusion

Cause-specific diagnosis of SCM is challenging, with limited and inconsistent information available across different studies. In our study, by using the current traditional diagnostic testing approaches, the cause-specific diagnosis of SCM remained unknown in one-fourth of the episodes and was made presumptively in 36%. Infectious meningitis was the predominant cause. The prognosis for SCM was poor, with 41% of cases resulting in an unfavourable outcome at hospital discharge, including a 14% in-hospital mortality rate. Factors significantly associated with higher mortality included older age, an immunocompromised state, the presence of the classic triad of meningitis, altered consciousness on presentation, severe hypoglycorrhachia, and low cerebrospinal fluid (CSF) leukocyte counts (<50/μL). Our study also revealed that patients with SCM of unknown cause had a significantly higher risk of in-hospital death and unfavourable outcomes at discharge compared to those with a proven or presumptive etiology. Accordingly, better prognosis of SCM can potentially be achieved through increasing the proportion of patients with cause-specific diagnosis. More rapid, low-cost, and accurate tests for the identification of a broad range of pathogens are needed to maximize diagnostic yield.

## Data Availability

The raw data supporting the conclusions of this article will be made available by the authors, without undue reservation.
